# The Roots of Bioinformatics in Theoretical Biology

**DOI:** 10.1371/journal.pcbi.1002021

**Published:** 2011-03-31

**Authors:** Paulien Hogeweg

**Affiliations:** Theoretical Biology and Bioinformatics Group, Department of Biology, Faculty of Science, Utrecht University, Utrecht, The Netherlands; Philadelphia, United States of America

## Abstract

From the late 1980s onward, the term “bioinformatics” mostly has been used to refer to computational methods for comparative analysis of genome data. However, the term was originally more widely defined as the study of informatic processes in biotic systems. In this essay, I will trace this early history (from a personal point of view) and I will argue that the original meaning of the term is re-emerging.

## Early History: Bioinformatics, a Work Concept

In the beginning of the 1970s, Ben Hesper and I started to use the term “bioinformatics” for the research we wanted to do, defining it as “the study of informatic processes in biotic systems”. (Although several public sources [see below] trace the origin of the term to publications by us that appeared in 1978 [1], [2], in fact we were using it as early as 1970, proposing the definition above in an article in Dutch that is not generally accessible [3].)

It seemed to us that one of the defining properties of life was information processing in its various forms, e.g., information accumulation during evolution, information transmission from DNA to intra- and intercellular processes, and the interpretation of such information at multiple levels. At a minimum, we felt that that information processing could serve as a useful metaphor for understanding living systems. We therefore thought that in addition to biophysics and biochemistry, it was useful to distinguish bioinformatics as a research field (or what we termed a “work concept”).

Indeed, at the birth of molecular biology it was recognized that a central research theme should be how living systems gather, process, store, and use information [4]. This focus on concepts related to information is, for example, reflected in the terminology “genetic code”, the central dogma as the unidirectional flow of information, etc. A nice monograph entitled “From Deoxyribonucleic Acid to Protein: Transfer of Genetic Information” [5] summarized the state of the art in molecular biology before the “sequence age”, unraveling for me the essential processes that, at the time in genetics undergraduate texts, were buried in “bead genetics”. It seems that recently, after a dormant phase, such information-centric terminology has become more prevalent again (e.g., in terms of identifying a distinct research field [4] and focusing on such processes as sensing the environment [6] and dynamic phosphorylation and methylation codes [7], [8]).

We were embedded then within theoretical biology. At the time, after general systems theory [9], [10] had come and gone, theoretical biology was in a mild resurgence in acceptance. The series of books entitled “Towards a Theoretical Biology”, edited by Waddington [11] (reprints of which are underway), had appeared a few years earlier. In 1972, the main topic at a meeting organized by BSRC (Biological Science Research Council) Developmental Biology in collaboration with the Society for Experimental Biology was mathematical models of development.

Stuart Kaufman was there, presenting his work on random Boolean networks, which introduced the concept of large-scale transcription regulation networks and viewed a cell type as an attractor in a multidimensional dynamical system [12]. It is striking that in the year 2000, Huang and Ingber reintroduced these concepts to the experimental molecular biology community [13] and later beautifully illustrated their power by demonstrating alternative trajectories to neutrophil differentiation on the basis of temporal gene expression data of 2,773 genes [14].

At this same meeting, models and experiments in such areas as oscillatory enzyme dynamics (e.g., [15], [16]), positional information [17], and bi-stability in gene regulation [18] were presented and hotly discussed. Spatial pattern formation was one of the central topics, contrasting Turing systems [19] with gradient-based systems [17]. Francis Crick, who in that period published some papers on gradients in development [20], attended the meeting. Skeptical about the emphasis Turing Patterns were (still) receiving, Crick quoted Turing as saying in reaction to enthusiasm about his work: “Well, the stripes are easy but what about the horse part?” To go “for the horse part”, i.e., to go beyond pattern formation to multilevel models of development and morphogenesis, became one of the long-term goals of our nascent work concept “bioinformatics”.

Also at about that time, John Maynard Smith gave a lecture in Utrecht and posed a similar challenge with respect to evolutionary biology as Turing's challenge relative to developmental biology. While evolutionary models mainly dealt with invasion of mutants and changing allele frequencies, the question of how evolution leads to complex organisms was not addressed. As Maynard Smith expressed it: “As good evolutionary biologists we should go once a year to the zoo and visit the elephant. We should greet it and say ‘Elephant, I believe you got about by random mutation’”. To meet the challenge of a “constructive evolutionary biology” became another long-term goal of bioinformatics as we envisioned it.

Research in artificial intelligence at this time was exploring new representations of information processing systems, often inspired by biological systems, e.g. neural network models for learning and pattern recognition [21], [22], genetic algorithms [23] for optimization, “actors” [24] for semi-independent parallel processing, and “turtle geometry” [25], [26], demonstrating the power of an individual self-centered approach to generating and/or understanding more global structures.

We felt that the re-introduction of biologically inspired computational ideas back into biology was needed in order to begin to understand biological systems as information processing systems. In particular, a focus on local interaction leading to emergent phenomena at multiple scales seemed to be missing in most biological models.

At the time, molecular biology was of course not a heavily “data-driven” science, as it would become with the advent of massive sequencing projects. Indeed, data-driven science was looked down upon, both in molecular biology and in theoretical biology. However, data-driven research was being done in the more traditional parts of biology, ecology, and taxonomy. I had just finished a data collection survey on water plant vegetation in India, Czechoslovakia, and The Netherlands and had become dissatisfied by the local state of the art of data processing, which comprised shuffling large tables by hand. At the same time, pattern recognition methods had already been introduced as “numerical taxonomy” [27], as well as in ecology [28], [29]. Although modeling and pattern analysis were (and still often are) seen as separate endeavors, we felt that for bioinformatic research they were both needed and should be combined: first, to analyze patterns of variation at multiple levels in organisms; second, to detect emergent phenomena in models; third, to compare the outcome of such models with “real” data; and finally, and most profoundly, because the relationship between genotype, phenotype, behavior, and environment itself can be seen as a type of pattern recognition or pattern transformation [30], [31], and understanding these processes was the core of bioinformatic research.

In short, under the heading of bioinformatics we wanted to combine pattern analysis and dynamic modeling and apply them to the challenge of unraveling pattern generation and informatic processes in biotic systems at multiple scales.

## Bioinformatics before the Data Deluge

But what could actually be done given the scarcity of data and paucity of computing power?

In fact, many of the basic pattern analysis methods now used in bioinformatics were pioneered in the 1960s (for a nice historical overview see [32]) and further developed in the 1970s. However, with respect to methods and data it was still a matter of everyone for themselves, as no easy exchange was possible. A notable exception was, of course, the work of Dayhoff to make protein sequences available through the yearly printed atlases of protein sequences and structure (from [33] to [34]). Accordingly, we spent much time in developing BIOPAT, an integrated set of supervised and nonsupervised pattern analysis methods, though at the same time we strenuously argued that methods development was NOT what bioinformatics was about.

We used the pattern analysis methods to study both “real” data and data derived from modeling studies. Our questions revolved around relating patterns of variation at different levels of organization. This included a first foray into non-linear genotype/phenotype mapping [35], using the developmental “grammars” introduced by Lindenmayer [36], [37], to demonstrate that the pattern of variation at the level of the genotype (the developmental rules) and at the level of the phenotype (the generated “morphemes”) does not necessarily coincide (as implicitly assumed in phylogenetic studies based on morphological data). We developed cluster analysis methods with iterative character weighting [38] to tease apart intermingled patterns of variation. Thus we could, for example, untangle morphological variation due to lineage differences and due to polyploidy [38]. In hindsight, it is interesting to recall the surprise (and dismay of the editors) when we found that isozyme variation was not correlated with lineage but with climatic conditions [39]. The general expectation was that, the closer to the genome, the closer to the “real” evolutionary relationships.

In the 1970s and 1980s, not only were pattern analysis methods developed, but novel modeling formalisms also were actively explored. Nonlinear systems started to become analyzable due to computer modeling, and new developments, for instance phase plain analysis, bifurcation diagrams, and deterministic chaos, were linked to biological applications (e.g., the logistic growth model is a prototype for deterministic chaos [40]).

Moreover, event-based modeling formalisms were developed; most well-known is the Gillespie algorithm developed for simulating chemical kinetics [41]. Our interests being on information processing and micro-macro transitions (emergent phenomena), we focused on the use and development of modeling formalisms implementing local interactions. Thus, we introduced cellular automata as a modeling formalism in ecology [42] and evolution [43], and developed event-based, individual-oriented (now usually called agent-based) simulation approaches.

Because of the often surprising and counterintuitive results of such models, we emphasized a bottom-up modeling methodology. Instead of designing a model to explain a priori well-defined results, in such a bottom-up modeling methodology known (or assumed) basic interactions are implemented, and the resulting dynamics are analyzed in multiple ways and at multiple levels. If and only if various seemingly unrelated and unforeseen consequences of the model correspond to the modeled system, this gives truly novel insight (and confidence in the model) [44], [45]. To analyze such models, pattern analysis methods can be indispensable to relate the outcome of the models to “real” data. For example, this allowed us to demonstrate that the behavioral patterns, division of labor, and adaptation to the environment observed in bumble bee colonies were emergent properties of local interaction of simple entities that “do what there is to do” [46]–[48].

## Data-Driven Bioinformatics

I recall the excitement when, in 1982, the first European Molecular Biology Laboratory sequence tape was delivered. Typing in data (on punch cards) from the Dayhoff atlases was cumbersome, even though many aligned sequences were provided. But what to do with this “mess” of data? Just for fun, we clustered species on nucleotide and dinucleotide content. To our surprise (and actually, dismay), a more or less decent classification emerged! This, in spite of our mantra that simple “amounts” would not take us very far in biology and we needed to look at patterns/information. But now we were back in the situation of almost a decade before: people trying to make sense of data by shuffling it around and finding by “eye/hand” some optimal arrangement, now with respect to aligning sets of sequences.

By developing an iterative guide tree-based multiple alignment method [49], we opened up this rich resource for our bioinformatic research. We pursued our earlier themes of coding structures and genotype/phenotype mapping through the study of RNA primary and secondary structure. It is gratifying that some of the multiple coding issues we studied are now being re-examined and that patterns we gleaned from the sparse data available at that time are now being verified through large-scale data analysis and direct high-throughput experiments. For example, we found that selection pressure on mRNA is not only related to protein coding but also to its secondary structure [50], [51], and inferred that “synonymous” mutations are therefore not necessarily neutral. Recently [52], it was inferred that conflicting selection pressures on synonymous codon use suggest just such selection pressure on secondary structure. As another example, we showed that a common pattern in mRNA secondary structure was a loosely folded 5′end in eukaryotic mRNA [53], apparently to facilitate translation initiation, a finding that has now been firmly established [54]–[56].

Propelled by the exponential increase of sequence data, the term bioinformatics became mainstream in the late 1980s, coming to mean the development and use of computational methods for data management and data analysis of sequence data, protein structure determination, homology-based function prediction, and phylogeny. But the rich insights obtained from the massive sequencing projects, and the related bioinformatic analysis to unravel function and evolution, is not really the “roots of bioinformatics”, but rather the “trunk of bioinformatics”, and not the subject of this article.

## Back to the Future

In 2002, I received a surprising e-mail from Oxford University Press: “It appears that you may be responsible for the term ‘bioinformatics’. I am preparing an entry for the word in the Oxford English Dictionary, and in this connection am investigating its history. . .” This led to our 1978 papers on chaotic dynamics in ecological models [1], and genotype phenotype mapping in growth models [2] being credited as the source of the term (though, as noted, our usage of it dated back to 1970). But was our definition of bioinformatics as the study of informatic processes in biotic systems at multiple levels just an historical quirk, to be superseded by the common meaning of the term as denoting the development and use of computational methods for comparative analysis of genome data?

The set of fully sequenced genomes (including human) was expanding, and high-throughput “omics” data entered the field, adding new dimensions to data-driven comparative research. Organisms were no longer just a “bag of genes or proteins” but also, e.g., a “bag of transcriptomes”, “a bag of interactomes”, and “a bag of metabolomes”. Integrating these various data is a marvelous opportunity and great challenge for bioinformatics in whatever sense of the word!

Indeed, the insight has again taken hold that organisms are not just a bag full of anything, but rather complex dynamical systems, and that an understanding of their functioning requires dynamical modeling. Under the heading “systems biology”, modeling efforts have been revived, and some of these efforts reflect the problems and dilemmas encountered already in the 1970s. How far can models be simplified and still be relevant? (Recall Einstein's dictum that “models should be as simple as possible but not more so”.) How can models be sensibly scaled up so as to meet the complexity revealed by the genomic data and still be manageable? As was the case in the 1970s with respect to “whole ecosystem” modeling [57], scaling up to the “whole cell” level appears most feasible for energy flow models [58]–[61], while large-scale kinetic models often suffer from the “parameter curse”. (The parameter curse was known in the 1970s as the “Loch Ness monster syndrome” after the existence of the creature was “proven” through population modeling showing that a large super-predator was apparently missing.) One way out of this dilemma might be to use evolutionary models [62].

Individual-based (agent-based) bottom-up modeling is still rare, but the detailed agent-based models of cell division [63] and locomotion [64] of Odell and coworkers are promising examples. The latter paper contains a nice discussion contrasting such detailed modeling with much simpler models that might equally fit the data (even if possibly for the wrong reasons), stressing that the power of such detailed models is to reveal novel counterintuitive consequences of the modeled interactions, as well as the surprising bonus that if detailed local interactions are modeled, robustness with respect to parameter choice often ensues.

So what about the long-term goals we set for bioinformatics in the 1970s, i.e., what of the “horse part” and the “elephant”? Some progress has been made in modeling morphogenesis in a strict sense (the “horse part”), through the use of cell-based models that incorporate some of the physical properties of cells [65]. In particular, the simple but biophysically reasonable representation of a cell in the CPM modeling formalism [66], [67] allows the scaling up to “computing an organism” [68] (e.g., the life cycle of *Dictyostelium* [69], [70]). But, as Segel emphasized, “the importance of linking changing gene expression with cell movement means that this achievement (i.e., computing an organism) is not the beginning of the end but rather the end of the beginning” [68]. Indeed, there lies the current challenge.

Constructive models of evolution (“the elephant”) have progressed from studies on the evolutionary consequences of non-linear “physical” genotype/phenotype mapping as exemplified by RNA folding [71]–[74] to the evolved genotype/phenotype mapping in the form of metabolic networks [75], [76], regulatory networks [77]–[80], and chromosome organization [81]–[83], and in “virtual cells” [84], [85]. These models shed light on the evolution of robustness and evolvability, and the interplay between neutrality and selection. Interestingly, the surprisingly large gene content of common ancestors as inferred from phylogenetic analysis of fully sequenced genomes and the major role of gene loss in the differentiation of lineages (cf. [86]) appear to be “normal” features in constructive models of evolution (T. Cuypers and P. Hogeweg, unpublished data; [87]). A general conclusion that can be drawn from these studies is that the multi-level nature of biological systems makes the evolutionary process through mutation and selection “easier” because of self-organization at many levels. However, here again the outstanding challenge is the closer integration of what does evolve in the models to what did evolve in nature, as gleaned from the bioinformatic analysis of genomic data.

As I am writing this, a video of Nobel laureate Paul Nurse has been posted in the science supplement of the *Guardian* newspaper [88]. Emphasizing self-organization and the resulting counterintuitive results, he argues that the next “quantum leap” in biology will come through studying information processing in biological systems. I conclude by asserting that, whether bioinformatics in the wider sense of studying information processing in biotic systems is a quirk or a quantum leap, it is certainly a mighty interesting quest!
